# Custom order entry for Parkinson’s medications in the hospital improves timely administration: an analysis of over 31,000 medication doses

**DOI:** 10.3389/fnagi.2023.1267067

**Published:** 2023-12-21

**Authors:** Hooman Azmi, Lisa Cocoziello, Francis Ruzicka, Elana Clar, John Michael Pederson, Blessy Jacob, Jewell Thomas, Anthony Rocco, Mary Bobek, Lucy Pereira-Argenziano, Patrick Roth, Florian P. Thomas

**Affiliations:** ^1^Department of Neurosurgery, Hackensack University Medical Center, Hackensack, NJ, United States; ^2^New Jersey Brain and Spine Center, Hackensack, NJ, United States; ^3^Hackensack Meridian School of Medicine, Nutley, NJ, United States; ^4^Department of Neurology, Hackensack University Medical Center, Hackensack, NJ, United States; ^5^Superior Medical Experts, St. Paul, MN, United States; ^6^Department of Pharmacy, Hackensack University Medical Center, Hackensack, NJ, United States; ^7^Department of Patient Safety and Quality, Hackensack University Medical Center, Hackensack, NJ, United States; ^8^Department of Nursing Clinical Education, Hackensack University Medical Center, Hackensack, NJ, United States

**Keywords:** Parkinsons disease, hospitalization, medication delays, custom orders, complications, safety, timely administration

## Abstract

**Background:**

Patients with Parkinson’s disease (PD) are at increased risk for hospital acquired complications. Deviations from home medication schedules and delays in administration are major contributing factors. We had previously developed a protocol to ensure adherence to home medication schedules using “custom” ordering. In this study we are assessing the impact this order type may have on reducing delays in PD medication administration in the hospital.

**Material and methods:**

We reviewed 31,404 orders placed for PD medications from January 2, 2016 to April 30 2021. We evaluated the orders to determine if they were placed in a Custom format or using a default non-custom order entry. We further evaluated all orders to determine if there was a relationship with the order type and timely administration of medications. We compared medications that were administered within 1 min, 15 min, 30 min and 60 min of due times across custom orders vs. non-custom default orders. We also evaluated the relationship between ordering providers and type of orders placed as well as hospital unit and type of orders placed.

**Results:**

14,204 (45.23%) orders were placed using a custom schedule and 17,200 (54.77%) orders were placed using non-custom defaults. The custom group showed a significantly lower median delay of 3.06 minutes compared to the non-custom group (*p*<.001). Custom orders had a significantly more recent median date than non-custom default orders (2019-10-07 vs. 2018-01-06, *p*<0.001). In additional analyses, medication administration delays were significantly improved for custom orders compared to non-custom orders, with likelihoods 1.64 times higher within 1 minute, 1.40 times higher within 15 minutes, and 1.33 times higher within 30 minutes of the due time (*p*<0.001 for all comparisons).

**Conclusion:**

This is the largest study to date examining the effects of order entry type on timely administration of PD medications in the hospital. Orders placed using a custom schedule may help reduce delays in administration of PD medications.

## Introduction

Patients with Parkinson’s disease (PD), especially those in more advanced stages, often rely on complex medication regimens to maintain function and quality of life (QoL). Errors or delays in medication administration can have a significant negative impact for this group. The consequences are particularly pronounced when PD patients are admitted to the hospital where rigid medication schedules, lack of PD knowledge among hospital staff, and limited availability of PD medications on hospital formularies can lead to missed or delayed dosing, medication substitutions, or even administration of contraindicated medications (Derry et al., 2010; Chou et al., 2011; Cohen and Smetzer, 2015; Shin and Habermann, 2016; Lertxundi et al., 2017; Mucksavage and Kim, 2020). These errors increase complication rates and prolong hospital stays (Barber et al., 2001; Derry et al., 2010; Gerlach et al., 2012; Carlson et al., 2014; Cohen and Smetzer, 2015; Martinez-Ramirez et al., 2015; Crispo et al., 2016; Lertxundi et al., 2017; Cox et al., 2018; Margolesky and Singer, 2018; Yu et al., 2023). Delays in medication administration of even 15 min have been shown to result in negative outcomes for PD patients (Parkinson’s Foundation, 2022).

Attempts to address such errors and delays have had varied success across institutions. Nance et al. demonstrated how nursing alerts in the Electronic Medical Records (EMR) and educational programs could improve timing of medication administration (Nance et al., 2020). Skelly et al. compared outcomes for patients with PD when admitted to general units vs. specialized units and observed a decreased length of stay and fewer medication errors (Skelly et al., 2014). Hobson and colleagues utilized an email alert to notify the specialist team when a PD patient was brought in through the emergency room. The alerts resulted in early interventions to address medications, and other needs of the patients (Hobson et al., 2019). Previously we reported on the design and implementation of a protocol aimed at better adherence to home medication regimens for hospitalized patients with PD, with emphasis on using “custom” orders instead of non-custom hospital defaults (Azmi et al., 2019). We further reported on the protocol’s impact in increasing the use of custom orders as well as improving patients’ length of stay (Azmi et al., 2020).

Any attempt to tackle such safety gaps for people with PD, need not only address adherence to the home medication regimen, but also timely dosing. To evaluate the effect of our protocol on timely dosing, we evaluated PD medications orders placed over a 5-year period. Herein, we report on the analysis of these orders with specific attention to the differences between dosing due time vs. dose administered time.

## Materials and methods

Following IRB approval, a retrospective review of PD medication orders placed from January 2, 2016, to April 30, 2021, was conducted at a single site, at Hackensack University Medical Center. 31,404 doses were analyzed. The primary outcome measure was whether the type of order placed (custom vs. non-custom) had any effect on the timely dosing of PD medications. (Non-custom orders are those which the default schedules such as BID or TID are used, whereas custom orders use a manual entry of specific medication times, ideally to reflect the patient’s home regimen). Timely dosing of medication was measured by the difference in the time recorded when the medication was administered by the nurse vs. the actual due time. The absolute value of the time difference was used to compare the two groups. Comparisons of frequency of medications administered within 1 min of medication due time, were modeled using a mixed effects model. Comparisons of median differences in time to medication administration relative to the due time of medication administration were modeled using mixed effects quantile regression.

Additional analyses were conducted for subgroups of medications administered within 15, 30 and 60 min of due time, as well as subgroups of ordering providers and ordering unit clusters. Descriptive analysis of all order types was also conducted.

### Statistical analysis

The full statistical methods can be found in the Supplementary Material. Descriptive statistics are reported as mean ± standard deviation, median (interquartile range), or as count (percentage). Normality of data was cross-validated using standard tests (Anderson and Darling, 1954; Shapiro and Wilk, 1965; D’agostino and Pearson, 1973). Medication administration within 1 min of due time was analyzed as a binary event (yes vs. no) using Fisher’s exact test. Effect sizes from Fisher’s exact test are reported as odds ratios (ORs), along with 95% CIs computed using the Baptista-Pike method (Fisher, 1922). For subgroup analysis multivariable models were used which included the ordering unit, provider type, and time of medication orders as fixed effects.

Multivariable analysis using a hierarchical mixed effects quantile regression was used to model conditional median differences in time to medication administration between groups, adjusted for fixed covariates as well as clustering variables. Additionally, a standard mixed effects generalized linear model was used to compare the conditional mean differences between groups; A mixed effects generalized linear model with a logistic link function was also chosen to compare frequencies of medications administered within 1 min between groups.

Descriptive analyses of frequency of medications administered within discrete time windows by group was also provided; dichotomized time windows included medications administered within 15-, 30-, and 60-min relative to the medication due date/time.

Additional exploratory analyses were performed to evaluate differences in usage of custom orders over time and between provider types and unit clusters (ER, ICU, Medical, Surgical, Psychiatric, Other).

All analyses were performed in RStudio (Version 2022.12.0, Build 353) running on R version 4.2.2. Mixed effects quantile regressions were performed using the ‘lqmm’ package and other multivariable analyses were performed using the ‘lme4’ package (Geraci and Bottai, 2014; Bates et al., 2015).

## Results

### Custom ordering

We reviewed 31,404 PD medication doses. Medications consisted of different formulations of carbidopa-levodopa, carbidopa-levodopa-entacapone, pramipexole, and ropinirole. Of all medication orders, 14,204 (45.23%) were placed using a “custom” schedule (Custom group) and 17,200 (54.77%) were placed using non-custom default schedules (Non-Custom group) (Table 1).

**Table 1:** Summary of characteristics by group.

Variable	Custom (*N* = 14,204)	Non-custom (*N* = 17,200)	Value of *p*
Median order date	2019-10-07	2018-01-06	<0.001
Earliest order date	2016-01-04	2016-01-02	.
Latest order date	2021-04-30	2021-04-28	.
Unit cluster			
ER	339 (2.4%)	817 (4.8%)	<0.001
ICU	866 (6.1%)	1,098 (6.4%)	0.303
Medical	7,717 (54.3%)	10,292 (59.8%)	<0.001
Surgical	4,886 (34.4%)	4,465 (26.0%)	<0.001
Psychiatric	229 (1.6%)	297 (1.7%)	0.453
Other	167 (1.2%)	231 (1.3%)	0.205
Provider type			
APNP	1,597 (11.2%)	2,296 (13.4%)	<0.001
Physician	8,864 (62.4%)	11,478 (66.7%)	<0.001
Physician assistant	1,008 (7.1%)	966 (5.6%)	<0.001
R1	380 (2.7%)	185 (1.1%)	<0.001
R2	341 (2.4%)	126 (0.7%)	<0.001
R3	1,363 (9.6%)	1,269 (7.4%)	<0.001
R4	232 (1.6%)	246 (1.4%)	0.151
R5	252 (1.8%)	450 (2.6%)	<0.001
R6	71 (0.5%)	107 (0.6%)	0.174
R7	96 (0.7%)	59 (0.3%)	<0.001
R9	0 (0%)	17 (0.1%)	>0.999
*Null*	0 (0%)	1 (0.01%)	>0.999

**Figure 1:**
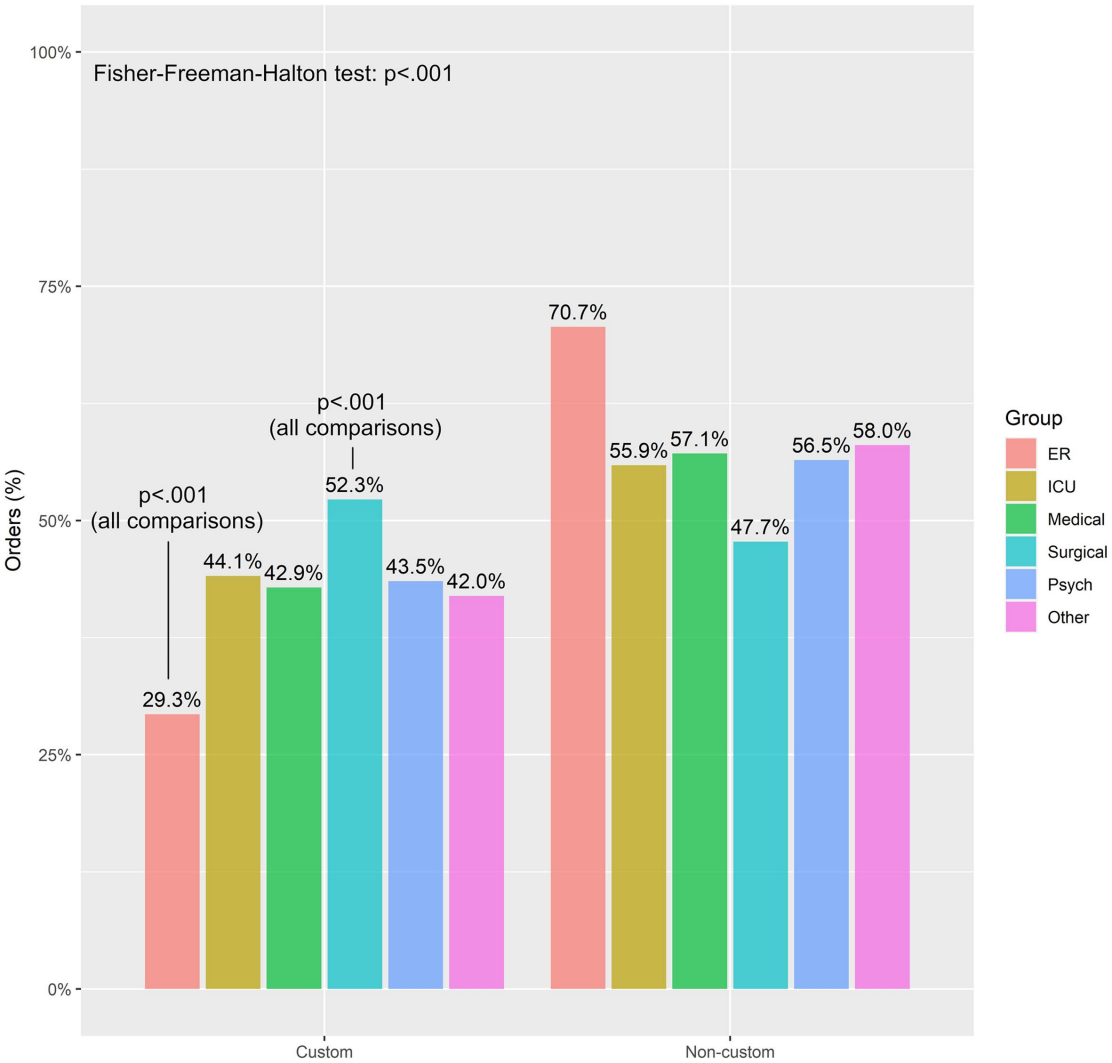
Frequency of custom and non-custom medication orders between unit clusters. ER tends to have the lowest amount of medication order, while surgical tends to have the highest amount of medication orders based on a Fisher’s exact test corrected for false discovery rate. ER, emergency room; ICU, intensive care unit.

There were no significant differences in the custom vs. the non-custom group when comparing orders placed in the ICUs, the Psychiatric unit or units grouped as “other.” (Units grouped as “other” were low census, non-surgical, non-medical units throughout the hospital). On the other hand, there were significantly more orders placed in the non-custom group in the ER and conversely more orders placed in the custom group in the Medical and Surgical units (Table 1). Within the custom group, orders rates were similar across the Medical [42.9% (7,717/18009)], ICU [44.1% (866/1964)], Psychiatric [43.5% (229/526)], and Other [42.0% (167/398)] units; however, the ER had significantly lower rate of custom orders [29.3% (339/1156)] while the Surgical unit cluster had a higher rate of these orders [52.3% (4,886/9351)] and was the only unit cluster to more commonly use custom medication orders over non-custom medication orders (Figure 1).

**Figure 2:**
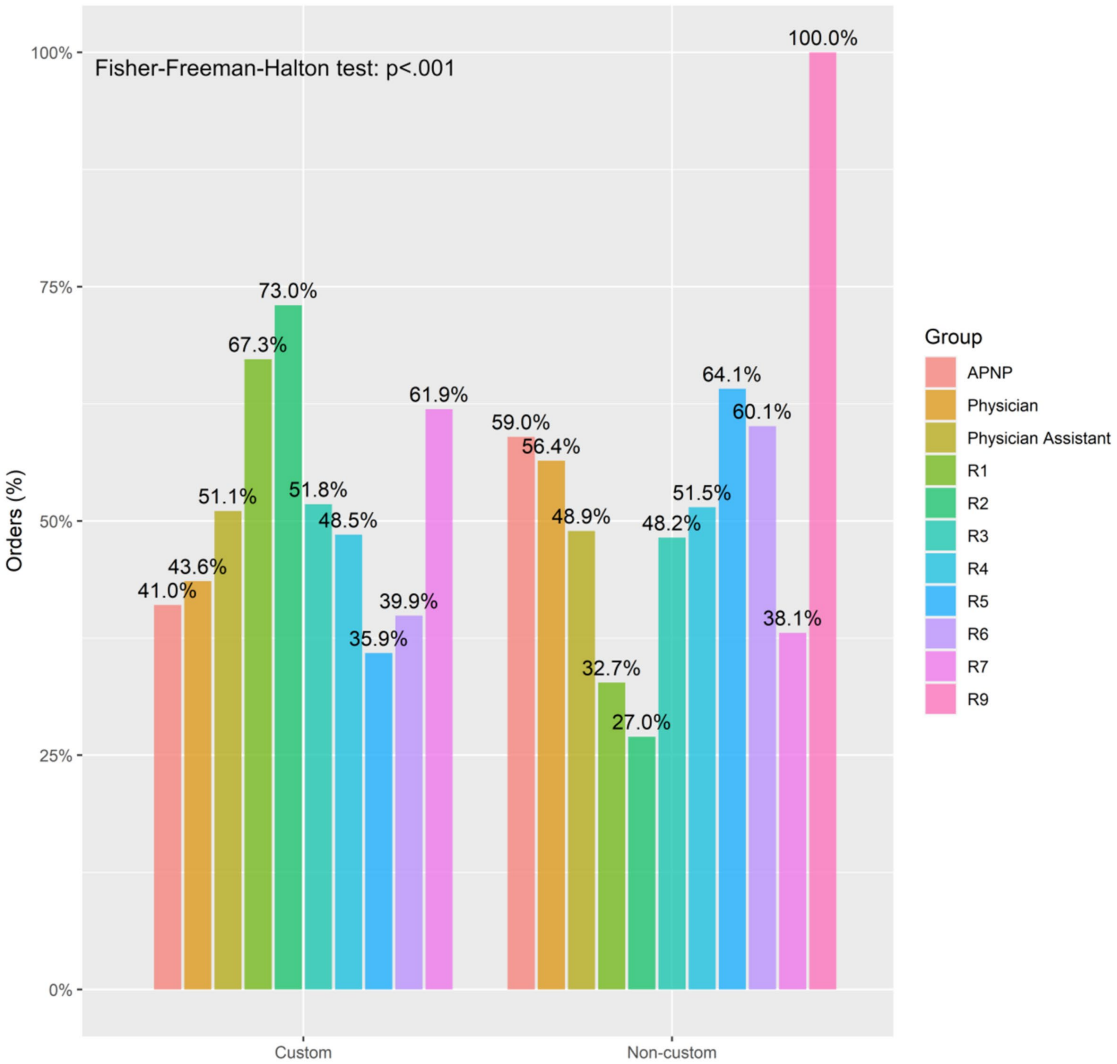
Frequency of custom and non-custom medication orders between medication provider types. APNP, advanced practice nurse practitioner.

The Custom group and Non-Custom groups had similar rates of medications ordered by year 4 resident (R4), year 6 resident (R6), and a year 9 resident (R9); however, the Custom group had a larger proportion of orders by physician assistants, year 1, 2, 3, and 7 residents (R1, R2, R3, R7) while the Non-Custom group had a larger proportion of orders by advanced practice nurses (APN), attending physicians, and year 5 residents (R5) (Figure 2).

**Figure 3:**
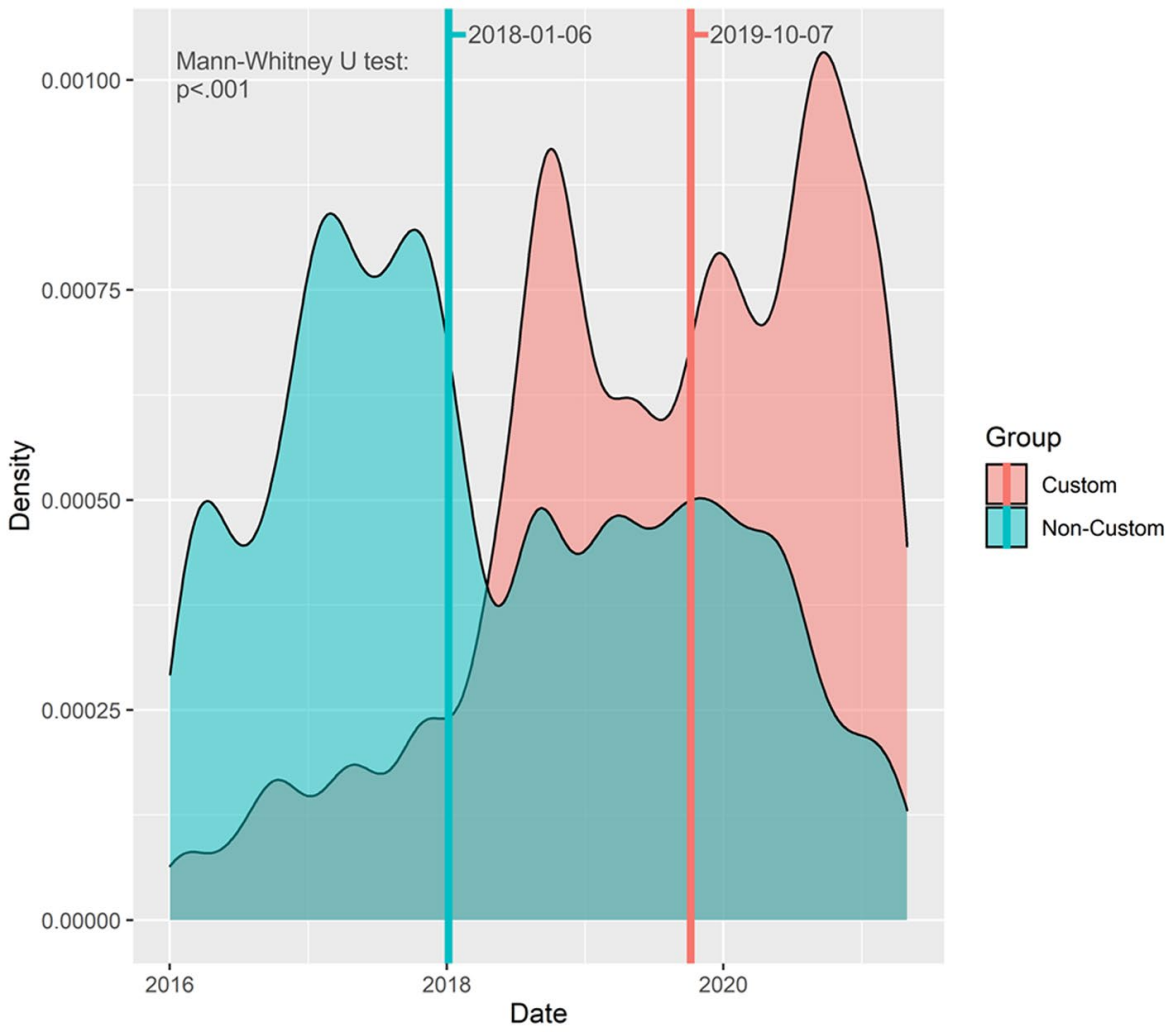
Density plot of ordered medication dates between groups. Lines showing the median values for each group are displayed, along with labels of the median values.

When comparing the entirety of orders across the study period, there was a significant difference in the median dates of orders in the custom group vs. those in the non-custom group. The Custom group tended to have more recent orders (median date = 2019-10-07) in comparison to the Non-custom group (median date = 2018-01-06); (Figure 3).

### Timely administration

The median difference in time to medication administration relative to the due time was 24 min (IQR: 9–51) in the Custom group compared to 29 min (IQR: 12–53) in the Non-Custom group. This difference was statistically significant, with a median difference of 3 min (95% CI, 3–4, *p* < 0.001).

When comparing the timing of medication between groups, 733 (5.16%) orders were administered within 1 min of due time in the Custom group compared to 522 (3.03%) in the Non-Custom group. This difference was statistically significant, with the odds of medications administered on time being 1.64 times as likely to occur in the Custom group compared to the Non-Custom group (95% CI, 1.55–1.95, *p* < 0.001; Table 2).

**Table 2:** Multivariable analyses of differences in time to medication administration between groups.

Outcome	Custom (*N* = 14,204)	Non-custom (*N* = 17,200)	Effect size	Value of *p*
Frequency of medications administered on time	733 (5.16%)	522 (3.03%)	1.67 (1.43–1.96)	<0.001
Median time difference relative to due time (minutes)	24 (9–51)	29 (12–53)	3.06 (1.48–4.46)	<0.001
Mean time difference relative to due time (minutes)	37.00 ± 46.25	40.57 ± 45.50	2.60 (1.01–4.18)	0.001

Effect sizes are reported as conditional odds ratios for “frequency of medications administered on time,” conditional median differences for “median time difference relative to due date/time” (from mixed effects quantile regression), and conditional mean differences for “Mean time difference relative to due date/time.” for each model, the non-custom is coded as the reference level; as such, the odds ratio of 1.67 implies that odds of medications being administered on time are 1.67 times as likely to occur in the Custom group compared to the non-custom group. Similarly, the median difference of 3.06, implies that the Custom group has a median decrease of 3.06 min in time to medication administration relative to the due date/time compared to the non-custom group. Models are adjusted for ordered date/time of medication, ADT unit, and medication provider type (see Supplementary Tables S1–S3 for detailed summaries showing the effect estimates for all variables included in models).

**Figure 4:**
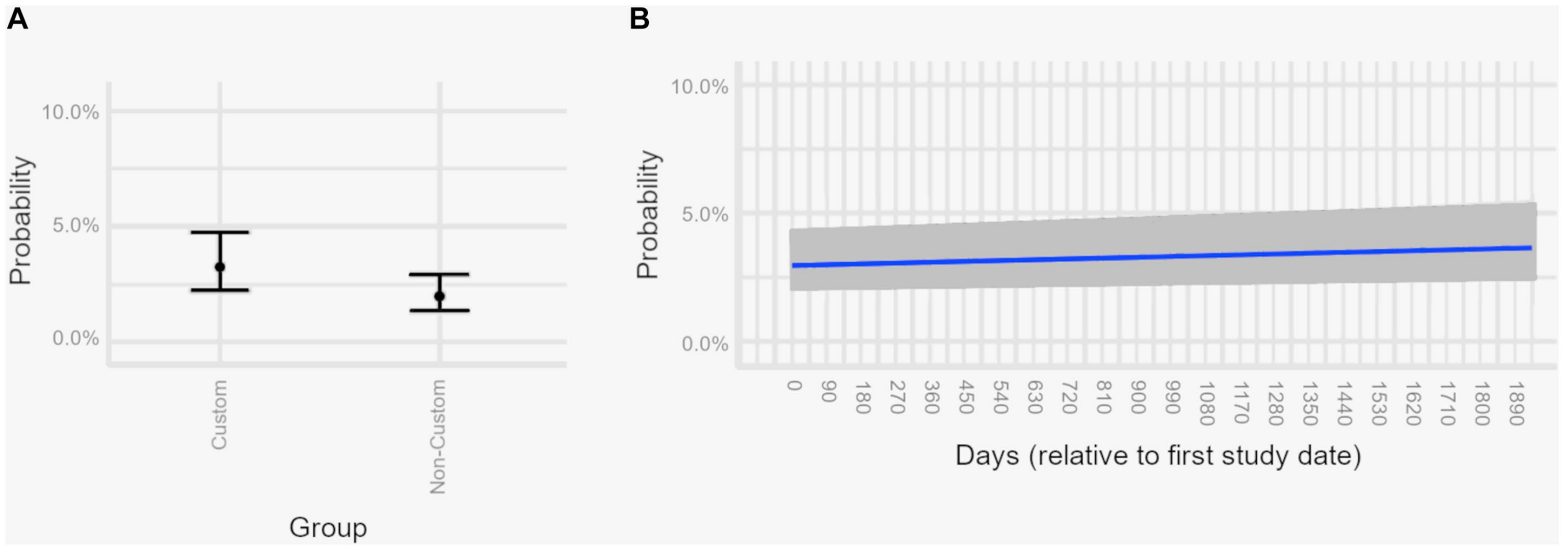
Estimated marginal effects plots, showing the predicted probabilities of medications administered on time across different predictor variables from a multivariable logistic regression model. Estimated marginal effects plots provide the predicted values for medications being administered on time at the margin of specific values (for continuous variables) or levels (for categorical variables), while holding non-focal variables constant and varying the focal variables. Medications are considered on time if administered within 1 min of the medication due date/time. Shaded areas and error bars represent 95% confidence intervals. Panel **(A)** Probability of any medication being administered within 1 min of due time if ordered “custom” vs. non-custom. **(B)** Probability of any medication being administered within 1 min of due time, demonstrating improvement over time.

Overall, when comparing the two groups of order types, the probability of the medication being administered within 1 min of due time was more when the medication order was “custom” compared to non-custom (Figure 4A). Moreover, the probability of any medication, regardless of order type, being administered within 1 min of due time increased over the study period (Figure 4B).

In addition to our focus on medications administered within 1 min of due times, we also compared subgroups of medications administered within 15 min, 30 min, and 60 min of due times to assess more practical subgroups.

**Figure 5:**
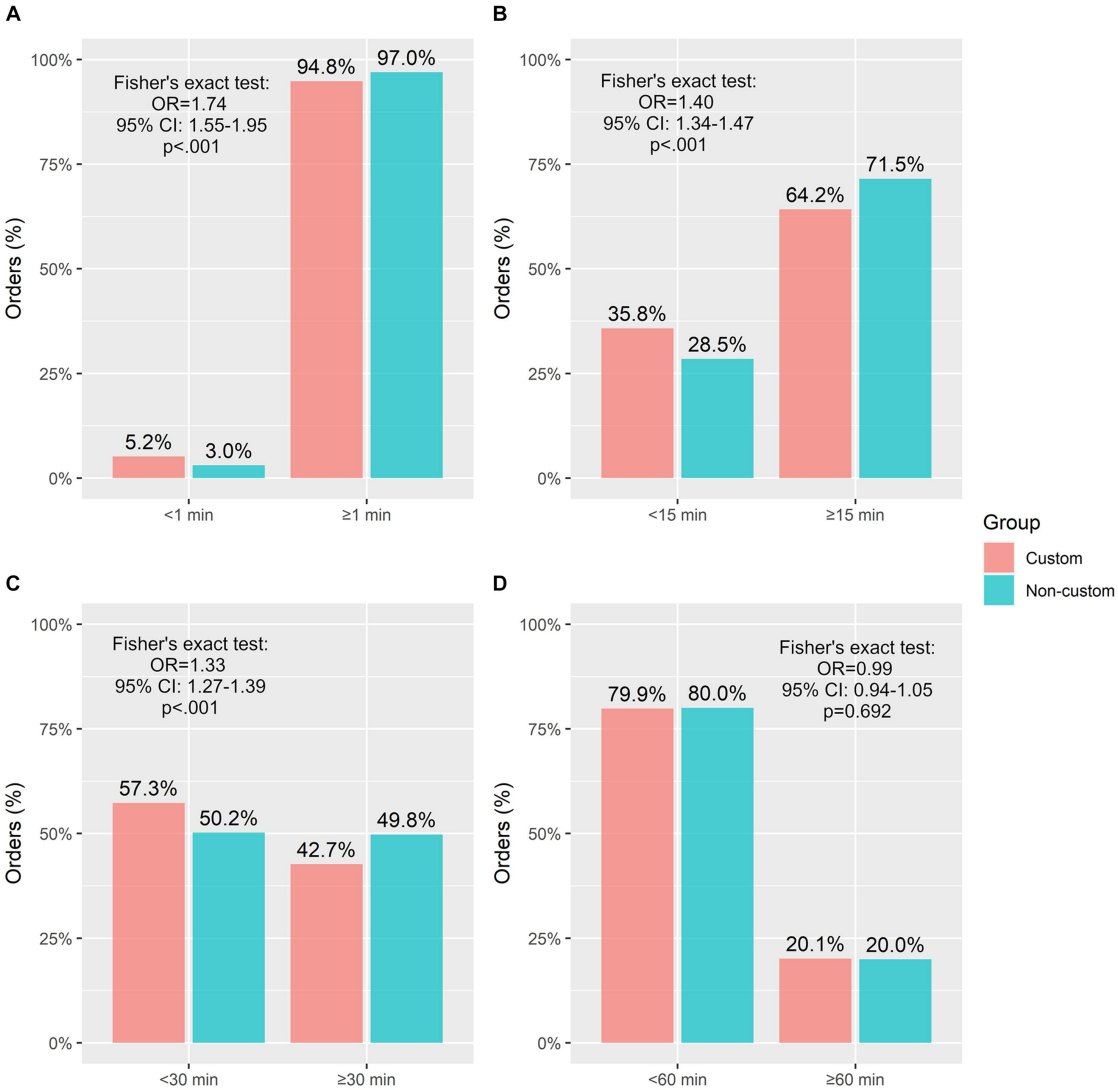
Frequency of medications administered within discrete time windows by group. **(A)** <1 min or ≥ 1 min, **(B)** <15 min or ≥ 15 min, **(C)** <30 min or ≥ 30 min, **(D)** <1 h or ≥ 1 h. OR, odds ratio; CI, confidence interval.

When orders were placed using a Custom format, it was 1.4 times more likely for the medication to be administered within 15 min of due compared to when the order was placed using a non-custom default (95% CI: 1.34–1.47, *p* < 0.001). Similarly, when medications orders were placed in a custom fashion, it was 1.33 times more likely for the medication to be administered within 30 min of due time compared to non-custom ordered medications (95% CI: 1.24–1.39, *p* < 0.001). This effect was not observed when medications were administered within 60 min from due time (Figure 5).

## Discussion

Timely administration of PD medications is critical to maintaining the safety of PD patients in the hospital. There is growing literature regarding worsening outcomes, delays in discharge and increased mortality with poorly managed PD medications in the hospital. Efforts to mitigate these issues by several institutions have demonstrated some positive potential, although widespread and universally effective processes have been lacking.

The fundamental challenge in achieving more uniform and comprehensive protocols is the lack of standardized guidelines. The Parkinson’s Foundation, one of three main advocacy groups for people with PD, has been actively creating awareness around these safety gaps. They recently put forth their Hospital Care Recommendations^1^ (Parkinson’s Foundation, 2023) as a tool for institutions to improve quality and safety of people with Parkinson’s in the hospital, which hopefully can serve as a steppingstone to development of national guidelines.

This study, To our knowledge, is the largest study to date to examine the effect of Custom order placement in the hospital on the timely administration of PD medications. The analysis did not exclude any hospital units with the objective of capturing the most accurate representation of delays in the administration of PD medications.

It is reassuring to see the findings demonstrate an increase in order placement using “Custom” schedules over time. This validates our educational process and our efforts to encourage adherence to the PD medication protocol, which was officially launched in 2018, as over time we see an increased likelihood of use of “custom” schedule ordering.

This analysis also supports the importance of placing PD orders using “Custom” ordering. Adherence to patients’ home medication not only helps reduce the risk of hospital acquired complications and decreases length of stay but may also positively impact timely administration of medication.

While the median administration delay for both groups was under 30 min, the custom group had 3 min less delay when comparing all doses across the time period analyzed. The comparison across subgroups demonstrated medications placed in a custom format were more likely to be administered within 1 min of due time. The same effect was seen when comparing medications given within 15 min of due time, as well as those given within 30 min of due time. The differences were statistically significant. These are powerful observations as there is evidence in the literature that even a 15-min delay in administration of PD medications can be deleterious for PD patients. While encouraged by the findings, clearly there is more work to be done as the majority of our medications were administered with over a 15-min delay.

The 60-min subgroup did not demonstrate a difference across custom and non-custom groups. This may be because this subgroup reflects a cohort where the administering nurses were unaware or unable to administer the medications in a timely manner reflected by the degree of delay, and as such the method of order placement may have been irrelevant.

When comparing order entry in various patient units across the hospital, it appears that most units or unit groupings performed similarly except for two outliers. The ER lagged the rest of the hospital in orders placed using custom schedules and the surgical units fared significantly better than other units for the same metric. The former finding is not surprising, given the nature of emergency room visits where the presenting complaint takes precedence. The latter finding may be explained by the origins of our medication protocol as it was developed in an attempt to ensure proper care of post deep brain stimulation surgery (DBS) patients and later disseminated through the entire hospital, and as such at least earlier in the process, the concepts were more familiar to the surgical units.

When evaluating ordering providers, it seems that custom ordering was used more commonly among physician assistants, first, second, third year and seventh year residents while non-custom ordering was more common among advanced practice nurses, attending physicians and fifth year residents. While it is not a clear-cut picture, certain patterns can be gleaned: the timeline of our educational process could account for the pattern of residents earlier in their careers being more aware of the importance of custom order placement. And the differences observed in ordering patterns between physician assistants and other advanced practice providers may be related to how these separate groups are organized within our hospital system and with which patient units they are more associated.

### Limitations

This study is limited by its retrospective nature. While the findings are encouraging for those institutions aiming to correct the risks faced by people with Parkinson’s in the hospital, they need to be validated in a prospective multicenter study.

There is also the consideration that while this study demonstrates a correlation between custom order placement and improvement in timely administration, this does not prove causation. There may be factors such as overall staff education that help improve timely administration. While this is possible, confounding factors should affect both order type groups equally. What we do observe however is a significant improvement in timely administration of medications when orders are placed using Custom timed schedules vs. non custom default schedules, supporting a more causative phenomenon.

## Conclusion

To our knowledge, this is the largest study to date examining the effects of order entry type on timely administration of medication. Based on this review of 31,404 medication doses administered across all units of our hospital, orders placed using a Custom schedule, may help reduce delays in administration of Parkinson’s medications.

## Data availability statement

The raw data supporting the conclusions of this article will be made available by the authors, without undue reservation.

## Ethics statement

The studies involving humans were approved by Hackensack University Medical Center Institutional Review Board. The studies were conducted in accordance with the local legislation and institutional requirements. Written informed consent for participation was not required from the participants or the participants’ legal guardians/next of kin in accordance with the national legislation and institutional requirements.

## Author contributions

HA: Conceptualization, Data curation, Investigation, Writing – original draft, Writing – review & editing. LC: Resources, Supervision, Writing – review & editing, Data curation. FR: Writing – review & editing, Data curation. EC: Writing – review & editing, Conceptualization, Methodology, Visualization. JP: Writing – review & editing, Formal analysis, Software. BJ: Resources, Writing – review & editing, Data curation, Project administration. JT: Data curation, Writing – review & editing, Project administration, Resources. AR: Data curation, Resources, Writing – review & editing. MB: Writing – review & editing. LP-A: Resources, Writing – review & editing. PR: Resources, Writing – review & editing. FPT: Writing – review & editing, Resources, Supervision.

## Footnotes

^1^ https://www.parkinson.org/sites/default/files/documents/hospital-care-recommendations-april2023.pdf
