# Supplementary material for: Custom order entry for Parkinson’s medications in the hospital improves timely administration: an analysis of over 31,000 medication doses
Source: Front Aging Neurosci. 2023 Dec 21;15:1267067. doi: 10.3389/fnagi.2023.1267067 (PMC10768191; doi:10.3389/fnagi.2023.1267067)
Supplement: Supplementary file 1 [file Data_Sheet_1.docx]

# **Supplementary Methods**

# **Statistical Analysis**

## ***Hypothesis***

The primary statistical hypothesis motivating the design of this retrospective study is that PD patients that have medications ordered on a custom schedule (“Custom” group) tend to have their medications administered closer to the target due date/time in comparison to patients that have medications ordered on a standard, non-custom schedule (“Non-Custom” group).

## ***Outcomes***

The primary outcome of interest was the difference in time to medication administration relative to the due date/time of medication administration between the Custom group and the Non-Custom group. Medications that were administered early or late were treated as equivalently poor outcomes; as such, the absolute value of the difference in time to medication administration relative to the due date/time was used for outcome comparisons between groups.

### ***Descriptive Statistics, Diagnostic Tests, and Univariate Comparisons***

Descriptive statistics are reported as mean ± standard deviation, median (interquartile range), or as count (percentage). Normality of data was assessed using cross-validation with the D'Agostino-Pearson,^1^ Anderson-Darling test,^2^ and Shapiro-Wilk tests.^3^ P-values <.10 were considered suggestive of a departure from normality, and if a significant departure was detected for any test, comparisons were made using non-parametric hypothesis tests. In each case, data demonstrated significant departures from normality and the Mann-Whitney U test was used for simple comparisons of continuous data between groups.^4^ Effect sizes from the Mann-Whitney U test are reported as Hodges-Lehmann differences (i.e., median of differences).^5^ Frequency of medications administered on time was analyzed as a binary event (i.e., on time or not on time), and Fisher’s exact test was used to compare frequency of medications administered on time.^6^ Medications were considered to be on time if administered within 1 minute of the medication due date/time. Effect sizes from Fisher’s exact test are reported as odds ratios (ORs), along with 95% CIs computed using the Baptista-Pike method.

### ***Multivariable Analyses***

Multivariable analysis using a hierarchical mixed effects quantile regression was used to model conditional median differences in time to medication administration between groups, adjusted for fixed covariates as well as clustering variables (e.g., encounter count). Due to the heavy-tailed, skewed nature of time to medication administration, data were fitted using quantile regression which is robust to high-leverage outliers and skewed marginal distributions. Additionally, a standard mixed effects generalized linear model (GLM) with an identity link function was used to compare the conditional mean differences between groups; however, emphasis should be placed on the multivariable quantile regression since data are highly skewed. A mixed effects GLM with a logistic link function was also chosen to compare frequencies of medications administered on time between groups. Mixed effects quantile regressions are fit using Laplace approximation and mixed effects GLMs are fit using restricted effects maximum likelihood.

### ***Covariates***

The following prespecified covariates were considered for multivariable regression, along with rationales for inclusion in the regression models:

- *Encounter Count:* Data has a natural hierarchical structure, where medication orders are nested within distinct encounter counts, with variability in time to medication administration related to the encounter count. Encounter count was considered a random effect.
- *Ordered Date/Time:* Time of medication orders varied substantially, with the earliest medication placed on 01/02/2016 while the latest was placed on 4/30/2021. Possible changes in treatment standards and hospital workflow over time may be related to timeliness of medication administration.
- *ADT Unit*: The specific ADT unit that the medication orders were placed at could have an impact on time to medication administration due to differences in workflow and business of the unit at the time of medication orders and medication administration time. ADT unit was considered hierarchical, with individual units nested within the following unit clusters:
  - Emergency Room (ER)
  - Intensive Care Unit (ICU)
  - Medical
  - Surgical
  - Psychiatric
  - Other
- *Medication Provider Type:* The specific medication provider type (physician, physician assistant, etc.) could have an impact on time to medication administration due to differences in workflow and business of different provider types at the time of medication orders at each unit.

Other covariates in the dataset included the specific medication ordered (ordered drug name, dose, etc.) and exact frequencies of orders within the Non-Custom group, but the study team considered these variables to be inconsequential to estimating the effect of ordering group on time to drug administration. The same set of covariates was included for each regression model.

### ***Subgroups and Other Exploratory Analyses***

Additional subgroup comparisons of time to medication administration across each of the categorical variables defined above were performed using the Kruskal-Wallis test^7^ for continuous data or Fisher’s exact test with Freeman-Halton’s extension^8^ for categorical data. Post-hoc analyses adjusted for multiple comparisons were performed using Dunn’s test for continuous data or using a series of Fisher’s exact tests.

Descriptive analyses of frequency of medications administered within discrete time windows by group are also provided; dichotomized time windows included medications administered within 15-, 30-, and 60-minutes relative to the medication due date/time.

Additional exploratory analyses were performed to evaluate differences in usage of custom orders over time and between provider types and ADT units.

### ***Software***

All analyses were performed in RStudio (Version 2022.12.0, Build 353) running on R version 4.2.2. Mixed effects quantile regressions were performing using the *‘lqmm’* package^9^ and other multivariable analyses were performed using the *‘lme4’* package.^10^

**References**

1. D'Agostino R, Pearson E. Tests for departure from normality. Empirical results for the distributions of b2 and √ b1. *Biometrika*. 1973;60:613-622

2. Anderson T, Darling D. A test of goodness of fit. *Journal of the American Statistical Association*. 1954;49:765-769

3. Shapiro S, Wilk M. An analysis of variance test for normality (complete samples). *Biometrika*. 1965;52:591–611

4. Mann H, Whitney D. On a test of whether one of two random variables is stochastically larger than the other. *The Annals of Mathematical Statistics*. 1947;18:50-60

5. Hodges J, Lehmann E. Estimates of location based on rank tests. *Annals of Mathematical Statistics*. 1963;34:598-611

6. Fisher R. On the interpretation of χ2 from contingency tables, and the calculation of p. *Journal of the Royal Statistical Society*. 1922;81:87-94

7. Kruskal W, Wallis W. Use of ranks in one-criterion variance analysis. *Journal of the American Statistical Association* 1952;47:583-621

8. Freeman GH, Halton JH. Note on an exact treatment of contingency, goodness of fit and other problems of significance. *Biometrika*. 1951;38:141-149

9. Geraci M, Bottai M. Linear quantile mixed models. *Statistics and Computing*. 2014;24:461-479

10. Bates D, Mächler M, Bolker B, Walker S. Fitting linear mixed-effects models using lme4. *Journal of Statistical Software*. 2015;67:1-48
