# Supplementary material for: Custom order entry for Parkinson’s medications in the hospital improves timely administration: an analysis of over 31,000 medication doses
Source: Front Aging Neurosci. 2023 Dec 21;15:1267067. doi: 10.3389/fnagi.2023.1267067 (PMC10768191; doi:10.3389/fnagi.2023.1267067)
Supplement: Supplementary file 2 [file Data_Sheet_2.docx]

**Supplementary Tables**

**Table S1.** Detailed summary of a mixed effects GLM with a logistic link function evaluating the effect of multiple predictors on medications arriving on time.

| **Variable** | **Value** | **95% CI** | **p-value** |
| --- | --- | --- | --- |
| (Intercept) | 0.06 | 0.04 to 0.09 | <.001 |
| Group (ref=Custom) |  |  |  |
| Non-custom | 0.60 | 0.51 to 0.70 | <.001 |
| Provider (ref=APNP) |  |  |  |
| Physician | 1.15 | 0.91 to 1.46 | .241 |
| Physician Assistant | 1.29 | 0.92 to 1.80 | .144 |
| R1 | 1.05 | 0.55 to 2.03 | .877 |
| R2 | 0.67 | 0.32 to 1.38 | .275 |
| R3 | 1.00 | 0.68 to 1.46 | .984 |
| R4 | 0.64 | 0.34 to 1.20 | .166 |
| R5 | 0.74 | 0.42 to 1.32 | .313 |
| R6 | 0.52 | 0.15 to 1.78 | .295 |
| R7 | 0.59 | 0.15 to 2.32 | .447 |
| R9 | 4.90 | 0.53 to 45.51 | .162 |
| NULL | <0.01 | <0.01 to NA | .897 |
| Unit (ref=ER) |  |  |  |
| ICU | 0.78 | 0.52 to 1.16 | .220 |
| Medical | 0.65 | 0.48 to 0.89 | .007 |
| Surgical | 1.14 | 0.83 to 1.58 | .417 |
| Psych | 0.45 | 0.20 to 1.00 | .049 |
| Other | 2.17 | 1.23 to 3.82 | .007 |
| Time | 1.00 | 1.00 to 1.00 | .001 |

Values represent the relative odds of medication orders being delivered on time from a mixed effects GLM with a logistic link function. For categorical variables, comparisons are made relative to a reference group. For instance, the value of 0.60 for the Non-custom group means that the odds of medications being delivered on time for the Non-custom group were 0.60 times as likely as the Custom group (reciprocal=1.67).

**Table S2.** Detailed summary of a mixed effects quantile regression evaluating the effect of multiple predictors on medication delays.

| **Variable** | **Value** | **95% CI** | **p-value** |
| --- | --- | --- | --- |
| (Intercept) | 42.74 | 37.54 to 47.93 | <.001 |
| Group (ref=Custom) |  |  |  |
| Non-custom | 3.06 | 1.48 to 4.64 | <.001 |
| Provider (ref=APNP) |  |  |  |
| Physician | 1.53 | -1.89 to 4.96 | .372 |
| Physician Assistant | -0.38 | -5.51 to 4.75 | .882 |
| R1 | -2.30 | -7.36 to 2.76 | .365 |
| R2 | 3.40 | -2.05 to 10.04 | .190 |
| R3 | 5.33 | -1.59 to 12.26 | .128 |
| R4 | 5.45 | -1.03 to 11.94 | .098 |
| R5 | 2.95 | -0.85 to 6.75 | .125 |
| R6 | 2.12 | -3.51 to 7.75 | .453 |
| R7 | 10.39 | -4.89 to 25.66 | .178 |
| R9 | -0.18 | -7.94 to 7.59 | .964 |
| NULL | -1.77 | -5.33 to 1.79 | .323 |
| Unit (ref=ER) |  |  |  |
| ICU | -13.95 | -18.41 to -9.49 | <.001 |
| Medical | -11.99 | -16.80 to -7.18 | <.001 |
| Surgical | -17.35 | -22.37 to -12.33 | <.001 |
| Psych | -6.43 | -14.73 to 1.88 | .126 |
| Other | -11.52 | -18.24 to -4.81 | .001 |
| Time | -0.004 | -0.006 to -0.002 | <.001 |

Values represent estimated differences relative to the medication due time from a mixed effects multivariable quantile regression model. For categorical variables, comparisons are made relative to a reference group. For instance, the value of 3.06 for the Non-custom group means that the Non-custom group had a 3.06 minute greater median difference in delivery times relative to due time compared to the Custom group. For the continuous covariate, *Time*, the value of -0.004 means that for every increase in 1-day, there is a -0.004-minute median time difference relative to the due date.

**Table S3.** Detailed summary of a mixed effects GLM with an identity link function evaluating the effect of multiple predictors on medication delays.

| **Variable** | **Value** | **95% CI** | **p-value** |
| --- | --- | --- | --- |
| (Intercept) | 61.62 | 57.42 to 65.83 | <.001 |
| Group (ref=Custom) |  |  |  |
| Non-custom | 2.60 | 1.01 to 4.18 | .001 |
| Provider (ref=APNP) |  |  |  |
| Physician | 2.34 | -0.08 to 4.75 | .058 |
| Physician Assistant | -0.03 | -3.63 to 3.56 | .988 |
| R1 | -2.49 | -9.17 to 4.13 | .463 |
| R2 | 8.45 | 2.01 to 14.87 | .010 |
| R3 | 3.62 | -0.29 to 7.50 | .070 |
| R4 | 7.63 | 1.74 to 13.52 | .011 |
| R5 | -0.31 | -5.73 to 5.10 | .911 |
| R6 | -0.41 | -9.60 to 10.42 | .936 |
| R7 | 4.86 | -8.00 to 17.72 | .461 |
| R9 | -9.65 | -42.36 to 23.06 | .565 |
| NULL | -16.46 | -105.15 to 72.42 | .717 |
| Unit (ref=ER) |  |  |  |
| ICU | -25.76 | -29.55 to -21.97 | <.001 |
| Medical | -21.94 | -24.73 to -19.15 | <.001 |
| Surgical | -27.08 | -30.08 to -24.07 | <.001 |
| Psych | -21.59 | -29.11 to -14.02 | <.001 |
| Other | -17.51 | -23.89 to -11.13 | <.001 |
| Time | -0.003 | -0.005 to -0.001 | .002 |

Values represent estimated differences relative to the medication due time from a mixed effects GLM with an identity link function. For categorical variables, comparisons are made relative to a reference group. For instance, the value of 2.60 for the Non-custom group means that the Non-custom group had a 2.60 minute greater median difference in delivery times relative to due time compared to the Custom group. For the continuous covariate, *Time*, the value of -0.003 means that for every increase, there is a -0.003-minute median time difference relative for every 1-day increase.
